# Effective Hamiltonian for silicene under arbitrary strain from multi-orbital basis

**DOI:** 10.1038/s41598-021-86947-z

**Published:** 2021-04-07

**Authors:** Zhuo Bin Siu, Mansoor B. A. Jalil

**Affiliations:** Department of Electrical and Computer Engineering, National University of Singapore, Singapore, Singapore

**Keywords:** Spintronics, Topological matter, Two-dimensional materials

## Abstract

A tight-binding (TB) Hamiltonian is derived for strained silicene from a multi-orbital basis. The derivation is based on the Slater–Koster coupling parameters between different orbitals across the silicene lattice and takes into account arbitrary distortion of the lattice under strain, as well as the first and second-order spin–orbit interactions (SOI). The breaking of the lattice symmetry reveals additional SOI terms which were previously neglected. As an exemplary application, we apply the linearized low-energy TB Hamiltonian to model the current-induced spin accumulation in strained silicene coupled to an in-plane magnetization. The interplay between symmetry-breaking and the additional SOI terms induces an out-of-plane spin accumulation. This spin accumulation remains unbalanced after summing over the Fermi surfaces of the occupied bands and the two valleys, and can thus be utilized for spin torque switching.

## Introduction

Silicene is the silicon analog of the more established two-dimensional material graphene, and consists of a honeycomb lattice of silicon atoms. Having been synthesised in recent experiments^1–5^, silicene has elicited much theoretical interest due to its novel transport properties such as giant mangetoresistance^6^, chiral superconductivity^7^, and various Hall effects^8–12^. These transport properties have made silicene a promising candidate in prospective device applications. In contrast to graphene, the larger atomic radius of silicon results in a buckling of the crystal lattice^8^ which in turn leads to a relatively large spin–orbit interaction (SOI) induced band gap^8,13,14^. An out-of-plane electric field can modulate the band gap and switch silicene between different topological phases^14–17^. Besides a sizable SOI, silicene is also attractive for spintronics applications due to its relatively long spin-diffusion time^18–20^, large spin coherence length^18–21^, high Fermi velocity^4^, and potential ease of integration into current silicon-based CMOS technology. In this light, a silicene-based field effect transistor was recently demonstrated experimentally^22^.

In this work, we derive an effective tight-binding Hamiltonian for strained silicene which is useful for modeling the effect of strain engineering in silicene. Strain engineering^23–26^ is an emerging technique for manipulating the properties of primarily two-dimensional materials through the mechanical distortion of the crystal lattices. Experimentally, strain can be applied in a controlled manner via free suspension across trenches^27^ or by deposition on textured substrates^28^. Numerous ab initio studies have shown, for example, that strain can affect the mechanical^29,30^ and thermal properties^31,32^ properties of silicene. More relevant to the current work, *ab initio* calculations have also shown that strain can shift the Fermi energy, change the band gap^33^ and the spin splitting^34^, and modulate other aspects of the bandstructure^30,35^. In particular, strain can displace the positions of the Dirac points in reciprocal space and introduce a directional anisotropy to the dispersion relation near these Dirac points^36,37^.

Most theoretical studies on strained silicene have focused on bandstructure calculations based primarily on ab initio methods. Less attention was given on how the resulting bandstructure affects the transport properties of strained silicene. In the latter, a convenient method is to consider the linear expansion of the Hamiltonian around the Dirac points and approximate the effects of strain by a single strain-dependent vector gauge potential term^23,38^. Using this approach, Wang et al. studied the spin and valley-resolved transmission in a silicene system consisting of a number of alternating strained and unstrained segments subjected to an out-of-plane electric field^39^. The gauge potential term has also used to study the generation of spin, lattice pseudo-spin, and valley-polarized currents in strained silicene in the presence of electromagnetic barriers^40^ and circularly-polarized light^41^.

A more refined approach has been adopted recently in which the hopping parameters of the tight-binding Hamiltonian in a lattice distorted by applied strain are modulated. Forkohnezhad et al. showed that the distortion results in a directional anisotropy of the carrier velocity^42^. Subsequently, Li et al. utilized the Slater–Koster parameters^13,43^ to derive the hopping integrals between the various orbitals in strained silicene^44^. However, the parameters themselves were obtained under the assumption of a regular silicene honeycomb lattice possessing rotational symmetry^13^.

Here, we apply the above Slater–Koster framework for a distorted silicene lattice under arbitrary strain to derive the effective low-energy tight-binding (TB) Hamiltonian. A TB Hamiltonian provides a good balance between capturing the relevant physics while being simple enough for practical numerical calculations and analytical derivations for device-oriented applications. On one end of the spectrum, ab-initio calculations offer the highest degree of accuracy, but they are inaccessible to most researchers because of the computational hardware and software requirements as well as the long calculation times required. On the other end, using a strain-dependent gauge to approximate the effects of strain in low-energy linear-momentum Hamiltonians is the simplest approach to incorporating strain but misses out much of the relevant physics. In particular, inter-valley effects, which are important in the emerging field of valleytronics^45^, cannot be modeled by such Hamiltonians.

Our derivation uncovers additional spin–orbit interaction (SOI) terms which have been neglected previously. Based on the TB Hamiltonian, we show that these additional terms result in current-induced out-of-plane spin accumulation in the presence of an *in-plane* magnetization coupling. In the absence of strain and the corresponding lattice distortion, the spin accumulation vanishes. In the presence of strain, the resulting symmetry-breaking ensures that the spin accumulation remains finite after summing over the entire Fermi surface and over the different valleys. The finite out-of-plane component may thus be utilized for strain-induced spin torque switching in silicene.

## Hamiltonian derivation

### Spin-independent Hamiltonian

The basic idea in our derivation of the low-energy effective Hamiltonian is to reduce a four-orbital Hamiltonian comprising the *s*, $p_x$, $p_y$, and $p_z$ orbitals at each lattice site and the nearest-neighbour couplings between them (the ‘four-orbital model’), to an effective single-orbital TB Hamiltonian about the Dirac points^13^. This approach has two advantages: First, it partially offsets the loss of the physics due to the interplay between the different orbitals that would be missed if we had started off from a single-orbital TB model outright and only modified the TB hopping terms, as was done in^42^. The terms that are discarded in the drastic simplifications made in assuming rotational and reflection symmetry in the derivation of the singe-orbital Hamiltonian in Ref.^13^ subsequently used in^42^ can now be restored when we begin afresh from a four-orbital model and drop the assumptions of rotational and reflection symmetry. At the same time, it is often unnecessary to know in detail which specific atomic orbitals contribute to phenomena of interest in device-oriented applications, such as the charge and spin accumulation and transport. A second key advantage of our approach is that the simplification of a four-orbital model into an effective single-effective orbital model results in smaller 4-by-4 Hamiltonian matrices in the latter compared to the original 16-by-16 matrices (including the spin degree of freedom) that are more tractable for both analytical and numerical works. For analytical works, the eigenvalues and eigenvectors of a larger matrix have necessarily more complicated forms with many terms that are cumbersome to work with and obscure the essential physics. For numerical works, the time complexity and memory requirements of common numerical eigenvalue algorithms scale with the square of the matrix dimensions.

Our single-orbital Hamiltonian is expressed in terms of the basis states $|A\rangle$ and $|B\rangle$ denoting the effective single orbitals localized at an *A* and *B* lattice site, respectively. This can basically be regarded as a tight-binding Hamiltonian, which has proved useful for transport and current-induced spin accumulation calculations (see, for example, Refs.^46–51^).

**Figure 1:**
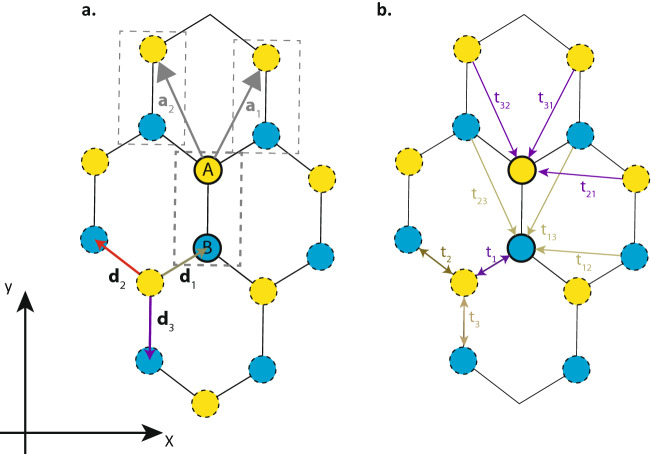
Schematic of a distorted silicene honeycomb lattice, lattice vectors, and inter-atomic couplings. (**a**) shows the *A* and *B* sublattice sites with bond displacement vectors $d_1$, $d_2$ and $d_3$ and nearest-neighbour coupling terms. The reference repeating unit cell, denoted by thicker boundaries, is connected to neighbouring unit cells by lattice translation vectors $\vec {a}_1$ and $\vec {a}_2$, which can be expressed in terms of the displacement vectors. (**b**) Schematic of the various coupling terms linking the first-neighbours and second nearest-neighbours for the lattice in (**a**).

Consider the distorted honeycomb lattice in Fig. 1 with a two-atom unit cell and the aforementioned four orbitals at each atom. We neglect the real spin degree of freedom for the time being, and will incorporate it in the next sub-section. The two atoms in each unit cell can be denoted as the *A* and *B* sublattice atoms. We denote the bond displacement vectors from an *A* sublattice site to its three nearest neighbours as ${\vec {d}}_1$ to ${\vec {d}}_3$, and define the primitive lattice vectors as

1 $$\begin{aligned} \vec {a}_1&= {\vec {d}}_1 - {\vec {d}}_3, \end{aligned}$$

2 $$\begin{aligned} \vec {a}_2&= {\vec {d}}_2 - {\vec {d}}_3. \end{aligned}$$

Owing to the lattice periodicity, the Hamiltonian can be written in the Bloch form of

3 $$\begin{aligned} H({\vec {k}}) = H_0 + \left( (\exp (i {\vec {k}}\cdot \vec {a}_1) H_1 + \exp (i {\vec {k}}\cdot \vec {a}_2) H_2) + {\text {h.c.}}\right) \end{aligned}$$

where *H* is a $(8\times 8)$ matrix (four orbitals per atom $\times$ two atoms per unit cell). In Eq. (3), the $H_i~(i=1,2)$s represent the hopping terms from atomic sites in the reference unit cell to those in the neighbouring unit situated at $\vec {a}_i$ away (see Fig. 1 ). $H_0$ contains the inter- and intra-site orbital coupling terms within the reference unit cell. The off-diagonal elements of *H* correspond to the hopping of the various orbitals between neighbouring lattice sites. $H_0$ takes the explicit form of

$$\begin{aligned} H_0 = \begin{pmatrix} \Delta &{} 0 &{} 0 &{} 0 &{} t^{ss}_3 &{} t^{sx}_3 &{} t^{sy}_3 &{} t^{sz}_3 \\ 0 &{} 0 &{} 0 &{} 0 &{} t^{xs}_3 &{} t^{xx}_3 &{} t^{xy}_3 &{} t^{xz}_3 \\ 0 &{} 0 &{} 0 &{} 0 &{} t^{ys}_3 &{} t^{yx}_3 &{} t^{yy}_3 &{} t^{yz}_3 \\ 0 &{} 0 &{} 0 &{} 0 &{} t^{zs}_3 &{} t^{zx}_3 &{} t^{zy}_3 &{} t^{zz}_3 \\ t^{ss}_3 &{} t^{sx}_3 &{} t^{sy}_3 &{} t^{sz}_3 &{} \Delta &{} 0 &{} 0 &{} 0 \\ t^{xs}_3 &{} t^{xx}_3 &{} t^{xy}_3 &{} t^{xz}_3 &{} 0 &{} 0 &{} 0 &{} 0 \\ t^{ys}_3 &{} t^{yx}_3 &{} t^{yy}_3 &{} t^{yz}_3 &{} 0 &{} 0 &{} 0 &{} 0 \\ t^{zs}_3 &{} t^{zx}_3 &{} t^{zy}_3 &{} t^{zz}_3 &{} 0 &{} 0 &{} 0 &{} 0 \end{pmatrix} \end{aligned}$$

with the basis states comprising of the *s*, $p_x$, $p_y$, and $p_z$ orbitals on the *A* and *B* sublattice sites. $\Delta$ is the on-site energy difference between the *s* and *p* orbitals and $t^{\alpha \beta }_3$ denotes the hopping coefficients between the $\alpha$ and $\beta$ orbitals. The 3 in the subscript denotes that this hopping is between first-neighbour sites separated by ${\vec {d}}_3$. Analogous expressions hold for $H_1$ and $H_2$ that contain the hopping terms $t^{\alpha \beta }_1$ and $t^{\alpha \beta }_2$ denoting hoppings between neighbouring lattice sites separated by ${\vec {d}}_1$ and ${\vec {d}}_2$ respectively.

The $t_\gamma ^{\alpha \beta }$ hopping terms can be expressed in terms of the Slater–Koster integrals^43^ $V_\gamma ^{ss\sigma }$,$V_{\gamma }^{{{\text{sp}}\sigma }}$, $V_\gamma ^{pp\sigma }$, and $V_\gamma^{pp\pi }$, and the direction cosines between the lattice sites^13^:

$$\begin{aligned} t^{ss}_\gamma&= V^{ss\sigma }_\gamma ,\\ t^{sx}_\gamma&= l_\gamma V^{ss\sigma }_\gamma , \\ t^{xs}_\gamma&= -l_\gamma V^{ss\sigma }_\gamma , \\ t^{xx}_\gamma&= l_\gamma ^2 V^{ss\sigma }_\gamma + (1-l_\gamma ^2) V^{pp\pi }_\gamma ,\\ t^{xy}_\gamma&= l_\gamma m_\gamma (V^{pp\sigma }_\gamma - V^{pp\pi }_\gamma ),\\ t^{yz}_\gamma&= m_\gamma n_\gamma (V^{pp\sigma }_\gamma - V^{pp\pi }_\gamma ). \end{aligned}$$

In the above, $(l_\gamma ,m_\gamma ,n_\gamma ) \equiv (d_\gamma ^x,d_\gamma ^y,d_\gamma ^z) / r_\gamma$ where $d_\gamma ^x$ is the *x* component of ${\vec {d}}_\gamma$, and $r_\gamma = |{\vec {d}}_\gamma |$. The other $t^{\alpha \beta }_\gamma$ not explicitly given above can be found by permuting the indices. Each of the Slater-Koster integrals $V^{ss\sigma }_\gamma$,$V^{sp\sigma }_\gamma$, $V^{pp\sigma }_\gamma$, and $V^{pp\pi }_\gamma$ can in turn be approximately expressed in the form of $\alpha _1 r_\gamma ^{-\alpha _2} \exp (-\alpha _3 r_\gamma ^{\alpha _4})$, where $\alpha _i~(i=1,\dots ,4)$ are material-dependent parameters. In our numerical calculations, we assume the values of $\alpha _i$ for silicon as given in Ref.^52^.

At each given value of ${\vec {k}}$, $H({\vec {k}})$ has eight energy eigenvalues, of which the lower four ($|\tilde{\varphi }_1\rangle$ to $|\tilde{\varphi }_4\rangle$) correspond to hole-like states and the upper four ($|\tilde{\varphi }_5\rangle$ to $|\tilde{\varphi }_8\rangle$) correspond to particle-like states. Within the first Brillouin zone, there are two Dirac points that are related to each other by time-reversal symmetry. At each Dirac point, the highest-energy hole state ($|\tilde{\varphi }_4\rangle$) and the lowest-energy particle state ($|\tilde{\varphi }_5\rangle$) are degenerate in energy. The degeneracy of these states imply that any linear combination of them will also be an energy eigenstate. Because our objective is to obtain an effective Hamiltonian with a single orbital contribution at each site, the most natural basis states for that Hamiltonian would be the two linear combinations of $|\tilde{\varphi }_4\rangle$ and $|\tilde{\varphi }_5\rangle$ that are the most localized at the *A* and *B* sublattice sites, respectively. For the purpose of determining these linear combinations, we define the sublattice or pseudospin operator $T^z$:

4 $$\begin{aligned} T^z \equiv \sum _{ o \in (s,p_x,p_y,p_z)} (|o^{A}\rangle \langle o^{A}| - |o^{B}\rangle \langle o^{B}|) \end{aligned}$$

where the index *o* runs over the *s*, $p_x$, $p_y$ and $p_z$ orbitals, and $|o^{ (A/B)}\rangle$ corresponds to the *o*th orbital localized at the (*A*/*B*) sublattice site. We then define the a $(2\times 2)$ matrix ${\tilde{\mathbf {T}}^z}$ with elements ${\tilde{\mathbf{T}}^z}_{ab} = \langle a|T_z|b\rangle$ for $|a,b\rangle \in (|\tilde{\varphi }_4\rangle , |\tilde{\varphi }_5\rangle )$. (Note that the boldface notation refers to an operator in a matrix representation using a specific basis set. The boldface notation is dropped when we refer to an operator generally without reference to a specific matrix representation.) The eigenvector of ${\tilde{\mathbf{T}}_z}$ with the positive (negative) eigenvalue would then correspond to the linear combination of $|\tilde{\varphi }_4\rangle$ and $|\tilde{\varphi }_5\rangle$ that is the most localized at the *A* (*B*) sublattice site. This eigenvector is denoted as $|\phi _A\rangle$ ($|\phi _B\rangle$). We now consider the linear expansion of the four-orbital Hamiltonian $H({\vec {k}})$ in Eq. (3) about the Dirac point at ${\vec {k}}={\vec {k}}_0$. For simplicity, we focus on the terms associated with $H_1$ in Eq. (3) (the terms associated with $H_2$ would follow by analogy, while the terms associated with $H_0$ are independent of ${\vec {k}}$). We then have

5 $$\begin{aligned} \exp (i ({\vec {k}}_0 + \delta {\vec {k}})\cdot \vec {a}_1) H_1 + \text {h.c.}\approx & {} \exp (i{\vec {k}}_0\cdot \vec {a}_1) (1 + i\delta {\vec {k}}\cdot \vec {a}_1) H_1 + \text {h.c.} \nonumber \\&= (\exp (i{\vec {k}}_0\cdot \vec {a}_1)H_1 + \text {h.c.}) + \nonumber \\&i(\delta {\vec {k}}\cdot \vec {a}_1)\left( \exp (i{\vec {k}}_0\cdot \vec {a}_1) H_1 - \exp (-i{\vec {k}}_0\cdot \vec {a}_1)H_1^\dagger \right) . \end{aligned}$$

Considering the second term of the above, we introduce

6 $$\begin{aligned} h_1 \equiv i( \exp (i{\vec {k}}_0\cdot \vec {a}_1) H_1 - \exp (-i{\vec {k}}_0\cdot \vec {a}_1)H_1^\dagger ). \end{aligned}$$

Recall that $h_1$ is associated with the hopping between the reference unit cell and its neighbouring unit cells in the $\pm \vec {a}_1$ direction. We now project $h_1$ into our “single-orbital” states $\{|\phi _A\rangle , |\phi _B\rangle \}$, such that it has matrix elements $\langle \phi _l|h_1|\phi _m\rangle ,~(l,m)\in \{{\text {A}}, {\text {B}}\}$. The non-diagonal elements of $h_1$ ($l \ne m$) represent hoppings between different sublattice sites, and hence correspond to nearest-neighbour sites in the unit cells separated by $\pm \vec {a}_1$ from the reference unit cell. (As shown in Fig. 1a, a given atom sits on a different sublattice site from its three nearest neighbours.) The diagonal matrix elements of $h_1$ in turn, correspond to hoppings between a given lattice site and its second nearest-neighbours along the $\pm \vec {a}_1$ directions. Repeating the above for $H_2$, the expansion of the Hamiltonian in Eq. (3) in the neighbourhood of $\vec {k_0}$ is therefore given by

7 $$\begin{aligned} \langle \phi _l|H({\vec {k}}_0 + \delta {\vec {k}})|\phi _m \rangle \approx \langle \phi _l|H({\vec {k}}_0)|\phi _m\rangle + \left( (\delta {\vec {k}}\cdot \vec {a}_1) \langle \phi _l|h_1|\phi _m\rangle + (\delta {\vec {k}}\cdot \vec {a}_2) \langle \phi _l|h_2|\phi _m\rangle \right) ,\ l,m \in (\text {A},\text {B}), \end{aligned}$$

where, in analogy with $h_1$, we define $h_2 \equiv i( \exp (i{\vec {k}}_0\cdot \vec {a}_2) H_2 - \text {h.c.})$. Note that by projecting the four-orbital model Hamiltonian to the states $\{|\phi _A\rangle ,|\phi _B\rangle \}$, we obtain a $\left( 2\times 2\right)$ matrix with the basis states localized at the *A* and *B* sublattice sites. However, the matrix still comprises of the coupling terms $t_\gamma ^{\alpha \beta }$ which involve all four orbitals *s*, $p_x$, $p_y$, and $p_z$. We will now relate the above projected Hamiltonian with the single-orbital TB Hamiltonian in the basis of the *A* and *B* sublattice sites, which we denote as $H^{(1)}$. Considering the nearest and next-nearest couplings as shown in Fig. 1, the $(2\times 2)$ single-orbital TB Hamiltonian can be written as

8 $$\begin{aligned} H^{(1)}&= \begin{pmatrix} E_0 &{} t_3^* \\ t_3 &{} E_0 \end{pmatrix} \nonumber \\ & \quad + {} \sum _{i=\{1,2\}} \begin{pmatrix} \text {e}^{i{\vec {k}}\cdot \vec {a}_i}t^A_{3i} + \text {c.c.} &{} t_i^* \text {e}^{i{\vec {k}}\cdot \vec {a}_i} \nonumber \\ t_i e^{i{\vec {k}}\cdot \vec {a}_i} &{} \text {e}^{i{\vec {k}}\cdot \vec {a}_i}t^B_{i3} + \text {c.c.} \end{pmatrix} \nonumber \\ & \quad + {} \begin{pmatrix}\text {e}^{i{\vec {k}}\cdot (\vec {a}_1-\vec {a}_2)}t_{21}^A + \text {c.c.} &{} 0 \\ 0 &{} \text {e}^{i{\vec {k}}\cdot (\vec {a}_1-\vec {a}_2)}t_{12}^B + \text {c.c.} \end{pmatrix}. \end{aligned}$$

In the above, $t_i$ represents the (single-orbital) nearest-neighbour hoppings in the direction of ${\vec {d}}_i$, while $t^{A(B)}_{lm}$ represents the corresponding second nearest-neighbour hopping linking the *A* (*B*) sublattice sites in the reference and neighbouring unit cells. The subscript *lm* indicates the sequence of the hopping, first along the $\pm {\vec {d}}_l$ direction followed by hopping along the $\pm {\vec {d}}_m$ direction (see Fig. 1b for the schematics of the second nearest-neighbour hoppings). In equation (8), $E_0$ is the common eigenenergy of $|\phi _{\text {A}}\rangle$ and $|\phi _{\text {B}}\rangle$ at the Dirac point. We will now express the single-orbital hopping terms, $t_i$ and $t^{A,B}_{lm}$, in terms of their four-orbital counterparts $t_\gamma ^{\alpha \beta }$. To do this, we expand the single-orbital Hamiltonian $H^{(1)}$ in Eq. (8) for a small $\delta {\vec {k}}$ about the Dirac point, and make a term-wise comparison with the linear expansion of the projected four-orbital *H* in Eq. (7), i.e. we equate

9 $$\begin{aligned} \partial _{k_i}H^{(1)}\delta k_i = \sum _{l \in \{A,B\}} |l\rangle \langle \phi _l| (\partial _{k_i}H)\delta k_i |\phi _m\rangle \langle m| \end{aligned}$$

for each $k_i = (k_x,k_y)$ individually. $|A\rangle$ and $|B\rangle$ are the single-orbital basis states at the A and B lattice sites respectively. As an example, let us consider the off-diagonal term $t_i\exp (i{\vec {k}}\cdot \vec {a}_i)$ in equation (8). This term corresponds to the hopping between the *A* to *B* sublattice sites in neighbouring unit cells separated by $\vec {a}_i$. Expanding to linear order in $\delta {\vec {k}}$, we have $t_i\exp (i{\vec {k}}\cdot \vec {a}_i) \approx t_i\exp (i{\vec {k}}_0\cdot \vec {a}_i)(1+ i\delta {\vec {k}}\cdot \vec {a}_i)$. On the other hand, the corresponding linear expansion of the projected four-orbital Hamiltonian in Eq. (7) yields $(\delta {\vec {k}}\cdot \vec {a}_i) \langle \phi _{\text {B}}|h_i|\phi _{\text {A}}\rangle$. Equating the two terms involving $(\delta {\vec {k}}\cdot \vec {a}_i)$, we have

10 $$\begin{aligned} t_i = {\frac{{\langle \phi _{\text {B}}|h_i|\phi _{\text {A}}\rangle }}{{i \exp (i{\vec {k}}_0\cdot \vec {a}_i)}}}. \end{aligned}$$

The above method cannot yield the corresponding expression for $t_3$ because $t_3$ is not involved in the linear expansion. Instead, $t_3$ is obtained by noting that the degeneracy of the hole and particle states at the Dirac point ${\vec {k}}_0$ implies that the off-diagonal terms in $H^{(1)}$ must sum to zero there. This yields the expression

11 $$\begin{aligned} t_3 = -t_1\exp (i{\vec {k}}_0\cdot \vec {a}_1) - t_2\exp (i{\vec {k}}_0\cdot \vec {a}_2) . \end{aligned}$$

Next, we proceed to derive the diagonal terms in equation (8), which represent the second nearest-neighbour couplings. Linear expansion of the terms containing $t^A_{3i}$ and $t^A_{21}$ in $\delta {\vec {k}}$ around ${\vec {k}}_0$ yields (for brevity we neglect the complex conjugate terms)

$$\begin{aligned}{} & \left( \sum _{i=\{1,2\}} \exp (i{\vec {k}}\cdot {a}_i)t^{A}_{3i} \right) + t^{A}_{21} \exp \left[ i{\vec {k}}\cdot (\vec {a}_1-\vec {a}_2)\right] \\ &\quad {}\approx \left( \sum _{i=\{1,2\}} e_i t^{A}_{3i}\right) + e_1e_2^*t^{A}_{21} +\dots \\&\quad\quad i\left[ (\delta {\vec {k}}\cdot \vec {a}_1)(-e_1^*t^{A*}_{31}+e_1 t^{A}_{31}) + (\delta {\vec {k}}\cdot \vec {a}_2)(-e_2^*t^{A*}_{32}+e_2 t^{A}_{32}) +\dots \right. \\&\quad\quad\left. (\delta {\vec {k}}\cdot (\vec {a}_1-\vec {a}_2))(-e_1^*e_2 t^{A*}_{21} + e_1e_2^* t^{A}_{21}) \right] \end{aligned}$$

where $e_i \equiv \exp (i {\vec {k}}_0\cdot \vec {a}_i)$. All the coupling constants $t_{ij}^A$ have to be real in order to preserve time-reversal symmetry. Imposing this constraint, the terms that are linear in $\delta {\vec {k}}$ simplify to

12 $$\begin{aligned}{}&i \left[ (\delta {\vec {k}}\cdot \vec {a}_1) (2i~\text {Im}(e_1) t^{A}_{31}) + (\delta {\vec {k}}\cdot \vec {a}_1) (2i~\text {Im}(e_2) t^{A}_{32}) + \delta {\vec {k}}\cdot (\vec {a}_1-\vec {a}_2)(2i~\text {Im}( e_1e_2^*) t^{A}_{21}) \right] \nonumber \\&~~ = -\sum _{l\in \{x,y\}}2 \left( (i_1 t^{A}_{31} a_{1l} + i_2 t^{A}_{32} a_{2l} + i_{12} t^{A}_{21} (a_{1l}-a_{2l}) \right) \delta k_l \end{aligned}$$

where $a_{1l}$ refers to the *l*th-component of $\vec {a}_1$, $\delta k_l$ refers to the *l*th-component of $\delta {\vec {k}}$, etc. In the above, $r_k \equiv \text {Re}(e_k)$ for $k=\{1,2\}$, $r_{12} \equiv \text {Re}(e_1e_2^*)$, and $i_k$ and $i_{12}$ are analogously defined for their imaginary parts. By comparison, the linear expansion of $\langle \phi _{A}|H({\vec {k}})|\phi _{A}\rangle$ about ${\vec {k}}_0$ from the four-orbital model is given by (refer to Eq. (7))

13 $$\begin{aligned} \langle \phi _{A}|H({\vec {k}} + \delta {\vec {k}})|\phi _{A}\rangle \approx \sum _{l\in \{x,y\}} (a_{1l} \langle \phi _{A}|h_1|\phi _{A}\rangle + a_{2l} \langle \phi _{A}|h_2|\phi _{A}\rangle ) \delta k_l, \end{aligned}$$

where we have neglected the $\delta {\vec {k}}$-independent terms. Matching the coefficients of $\delta k_x$ and $\delta k_y$ in Eqs. (12) and (13) gives two equations for the three unknowns $t^{A}_{31}$, $t^{A}_{32}$ and $t^{A}_{12}$. A third equation is given by the requirement that the second nearest-neighbour terms vanish at the Dirac points. (Note that a finite second nearest-neighbour coupling will cause an energy displacement of the single-orbital Hamiltonian Dirac point with respect to that of the four-orbital Hamiltonian.) In addition, by symmetry, we have $\langle \phi _{A}|h_i|\phi _{A} \rangle = \langle \phi _{B}|h_i|\phi _{B} \rangle$, so that $t^{A}_{3i} = t^{B}_{3i} = t_{3i}$ and $t^{A}_{12} = t^{B}_{12} = t_{12}$. Solving the simultaneous linear equations, the second nearest-neighbour couplings of the single-orbital Hamiltonian are finally given by

14 $$\begin{aligned} \begin{pmatrix} t_{31} \\ t_{12} \\ t_{32} \end{pmatrix} = \begin{pmatrix} -2 i_1 a_{1x} &{} -2i_{12} (a_{1x} - a_{2x}) &{} -2 i_2 a_{2x} \\ -2 i_1 a_{1y} &{} -2i_{12} (a_{1y} - a_{2y}) &{} -2 i_2 a_{2y} \\ r_1 &{} r_{12} &{} r_{2} \end{pmatrix}^{-1} \begin{pmatrix} a_{1x} \langle \phi _{A}|h_1|\phi _{A}\rangle + a_{2x} \langle \phi _{A}|h_2|\phi _{A}\rangle \\ a_{1y} \langle \phi _{A}|h_1|\phi _{A}\rangle + a_{2y} \langle \phi _{A}|h_2|\phi _{A}\rangle \\ 0 \end{pmatrix}. \end{aligned}$$

### Spin–orbit interaction

In the previous section, we derived the single-orbital TB Hamiltonian from the four-orbital Hamiltonian based on the Slater–Koster integrals for the case of a spinless Hamiltonian. In this section, we perform the derivation in the presence of spin-dependent terms in the Hamiltonian. We incorporate the atomic spin–orbit interaction (SOI) Hamiltonian $H_{\text {SO}} = \xi _0 (\vec {L}\cdot {\vec {\sigma }})$ into the four-orbital Hamiltonian, where $\vec {L}$ is the orbital angular momentum operator, ${\vec {\sigma }}$ the spin operator, and $\xi _0$ is a scalar representing the SOI strength. Classically, $H_{\text {SO}}$ can be interpreted as the interaction energy of the electron spin magnetic moment with the magnetic field generated by the electron orbital motion. With the SOI term, the four-orbital Hamiltonian is now given by

15 $$\begin{aligned} H^{\text {S}(4)}({\vec {k}}) = H({\vec {k}})\otimes {\mathbf {I}}_{\sigma } + H_{\text {SO}}, \end{aligned}$$

where the $H({\vec {k}})$ is the spin-less four-orbital Hamiltonian discussed in the previous subsection, i.e., Eq. (3). In the four-orbital model, the atomic SOI Hamiltonian acts only on the orbitals within a single site. However, the combination of the atomic SOI with the inter-site Slater–Koster orbital hoppings results in SOI terms that couple different lattice sites in the single-orbital Hamiltonian. In the case of unstrained silicene, $H_{\text {SO}}$ results in two SOI terms in the single-orbital model: (1) a $\delta {\vec {k}}$-independent out-of-plane SOI term and (2) an intrinsic Rashba SOI with the form $(\delta {\vec {k}}\times \hat{z})\cdot {\vec {\sigma }}$ in the vicinity of a Dirac point.

We consider the expression of $H_{\text {SO}}$ in the four-orbital model. Note that only the $p_{x,y,z}$ orbitals contribute to $H_{\text {SO}}$. Denoting the $p_i$ orbital ($i\in \{x,y,z\}$) at lattice site *a* with spin *s* as $|p_i^a,s\rangle$, $H_{\text {SO}}$ can be succinctly expressed as $H_{\text {SO}} = -\sum _{a\in \{A,B\}}{\frac{i}{2}}\epsilon _{i,j,k}\xi _0 |p_i^a,s\rangle \sigma ^k_{s,s'} \langle p_j^a,s'|$, where $\epsilon _{i,j,k}$ is the Levi–Cevita symbol.

Next, as in the previous subsection, we project the above four-orbital $H_{\text {SO}}$ to the states $\{|\phi _A\rangle ,|\phi _B\rangle \}$ which are closest to the Dirac point and localized at the *A* and *B* sublattice sites, respectively. This time, however, we need to include the spin degree of freedom. We introduce $|\phi _{\text {A}}, \uparrow \rangle \equiv |\phi _{\text {A}}\rangle \otimes |\uparrow \rangle$ where $|\uparrow \rangle$ is the spinor state with spin in the $+z$ direction. (The $|\phi _{\text {A}/\text {B}}\rangle$s are the same $|\phi _{\text {A}/\text {B}}\rangle$s as in the previous subsection, i.e. the Dirac point eigenstates localized at the A/B sublattice sites without the SOI.) Analogously, the other three combinations are written as $|\phi _{A(B)},\uparrow (\downarrow )\rangle$. We consider the first and second-order SOI terms which are proportional to $\xi _0$ and $\xi _0^2$, respectively. In unstrained silicene, the first and second-order SOI terms contribute to the $\delta {\vec {k}}$-independent SOI term, while the second-order SOI term contributes to the intrinsic Rashba SOI.

Projecting $H_{\text {SO}}$ into the set of four $|\phi _{A (B)}, \uparrow (\downarrow )\rangle$ states at the Dirac point ${\vec {k}}_0$, we obtain the first-order SOI term

16 $$\begin{aligned} H^{(1)}_{({\text{SO}}; 1)}({\vec {k}}_0) = \sum _{l,m\in \{A,B\}} \sum _{s,s'=(\uparrow ,\downarrow )} |l, s \rangle \langle \phi _l,s|H_{\text {SO}} ({\vec {k}}_0)|\phi _m,s'\rangle \langle m, s'|. \end{aligned}$$

The superscript “(1)” indicates that the Hamiltonian is a single-orbital model, while the subscript “1” denotes the first-order SOI term. Note that in the above, the ket vector $|l,s\rangle$ represents the single-orbital basis states, with $l\in \{A,B\}$ denoting the sublattice index and $s\in \{\uparrow ,\downarrow \}$ the spin index (and similarly for the bra vector $\langle m,s'|$). The term $\langle \phi _l,s|H_{\text {SO}} ({\vec {k}}_0)|\phi _m,s'\rangle$ denotes the inner product of $H_{\text {SO}}$ with the four-orbital states $|\phi _m,s\rangle$ states. As abovementioned, the $|\phi _m,s\rangle$ states are largely localized at the $m\in \{A,B\}$ sublattice site, and contains the four-orbital hopping integrals. In practice, the off-diagonal terms of $H^{(1)}_{({\text{SO}}; 1)}$ are small and can be ignored to a first approximation, and the diagonal terms have equal magnitudes but opposite signs. Hence, $H^{(1)}_{({\text{SO}}; 1)}\propto \tau _z$ , where $\varvec{\tau }=(\tau _x,\tau _y,\tau _z)$ denotes the vector of Pauli matrices in the pseudospin (sublattice) degree of freedom where $\tau _z = |A\rangle \langle A| - |B\rangle \langle B|$, and $\tau _x$ and $\tau _y$ can be defined analogously. We can then express $H^{(1)}_{(\text {SO};1)}({\vec {k}}_0)$ at the Dirac point ${\vec {k}}_0$ as

17 $$\begin{aligned} H^{(1)}_{(\text {SO};1)}({\vec {k}}_0) \approx \sum _{s\in \{x,y,z\}} h_{s;1} \sigma _s \otimes \tau _z \end{aligned}$$

where $h_{s;1}$ denotes the first-order SOI coefficients associated with spin *s* at ${\vec {k}}={\vec {k}}_0$. These coefficients can be extracted via the relation $h_{s;1} = {\text {Tr}} ( (\tau _z\otimes \sigma _s) H^{(1)}_{(\text {SO};1)}) / 4$. In unstrained silicene, $H^{(1)}_{(\text {SO};1)}$ contributes to the $\delta {\vec {k}}$-independent, out-of-plane SOI term in the linear expansion of the Hamiltonian around the vicinity of the Dirac points. At first sight, it would appear that $H^{(1)}_{(\text {SO};1)} ({\vec {k}}_0)$ corresponds to a constant exchange energy term in the single-orbital model, i.e., one having the form of ${\vec {m}}\cdot {\vec {\sigma }}$ where ${\vec {m}}$ is *k*-independent. Such a term is however prohibited by time-reversal symmetry, which reverses the sign of ${\vec {\sigma }}$. To preserve time-reveral symmetry, $H^{(1)}_{(\text {SO};1)} ({\vec {k}}_0)$ should instead be interpreted as the value of a *k*-*dependent* SOI term with an odd dependence on ${\vec {k}}$ at the specific value of ${\vec {k}}={\vec {k}}_0$. Because $H^{(1)}_{(\text {SO};1)}$ is diagonal in the sublattice degree of freedom, hence, from Fig. 1, the SOI term would comprise of second nearest-neighbour coupling terms. The ${\vec k}$-dependence of the single-orbital SOI Hamiltonian at ${\vec {k}}\ne {\vec {k}}_0$ will be addressed after we introduce the second-order SOI term.

The second-order SOI term, $H^{(1)}_{(\text {SO}; 2)}$ accounts for the effect of the atomic $H_{\text {SO}}$ on all the eight eigenstates of $H({\vec {k}})$ in equation (3) . It can be interpreted as an additional term added to the single-orbital Hamiltonian to capture some of the SOI physics that would otherwise be lost in simplifying the four-orbital model to a single-orbital one. At the Dirac point ${\vec {k}}={\vec {k}}_0$, the eigenstates of $H({\vec {k}}=\vec {k_0})$ include $|\phi _{\text {A}}\rangle$ and $|\phi _{\text {B}}\rangle$ which were introduced previously, as well as six others, some of which may be pair-wise degenerate. For these degenerate pairs, we express them as linear combinations which would diagonalize $T_z$, similar to the treatment in obtaining $|\phi _{\text {A}}\rangle$ and $|\phi _{\text {B}}\rangle$. Let us label the other six eigenstates as $|\varphi _i\rangle$, $i\in \{1,2,...,6\}$. We introduce the spin degree of freedom: $|\varphi _i,\sigma \rangle ,~\sigma \in \{\uparrow ,\downarrow \}$, and define a $(12\times 12)$ matrix $H_{\text {OO}}({\vec {k}})$ with elements $\langle \varphi _i,\sigma |H ({\vec {k}})\otimes {\mathbf {I}}_\sigma |\varphi_j,\sigma '\rangle$, $i,j\in \{1,2,\dots ,6\}$, and a $(12\times 4)$ matrix $H_{\text {OD}}({\vec {k}})$ with elements $\langle \varphi _i,\sigma |H ({\vec {k}})\otimes {\mathbf {I}}_\sigma |\phi _j,\sigma '\rangle$, $i \in \{1,2,\dots ,6\}, j \in \{\text {A},\text {B}\}$. (The “D” in the subscript denotes the four states at the Dirac points, i.e., $|\phi _{\text {A}}, (\uparrow /\downarrow ) \rangle$ and $|\phi _{\text {B}}, (\uparrow /\downarrow ) \rangle$, while the “O” in the subscript denotes the other states.) The second-order SOI Hamiltonian $H^{(1)}_{({\text{SO}}; 2)}({\vec {k}})$ of the single-orbital model is then obtained from Löwdin partitioning^13^ and is given by

18 $$\begin{aligned} H^{(1)}_{({\text{SO}}; 2)}({\vec {k}}) = - H_{\text {OD}}^\dagger ({\vec {k}}) (H_{\text {OO}}({\vec {k}}) - E_0 )^{-1} H_{\text {OD}}({\vec {k}}). \end{aligned}$$

Expanding $H^{(1)}_{({\text{SO}};2)}({\vec {k}}_0+\delta {\vec {k}})$ to linear order in $\delta {\vec {k}}$ around the Dirac point, we have

19 $$\begin{aligned} H^{(1)}_{({\text{SO}};2)}({\vec {k}}_0 + \delta {\vec {k}} ) \approx H^{(1)}_{({\text{SO}};2)}({\vec {k}}_0) + \sum _{l\in \{x,y\}}\big ( \partial _{k_l} H^{(1)}_{({\text{SO}};2)}(\vec {k_0})\big ) \delta k_l. \end{aligned}$$

$H^{(1)}_{(\text {SO;2})}({\vec {k}}_0)$ and $\partial _{k_l} H^{(1)}_{(\text {SO};2)} ({\vec {k}}_0)$ are evaluated numerically using equation (18) and the finite difference approximation $\partial _{k_l} H^{(1)}_{(\text {SO};2)} ({\vec {k}}_0) \approx (H^{(1)}_{(\text {SO};2)} ({\vec {k}}_0 + \delta _k \hat{e}_l) - H^{(1)}_{(\text {SO};2)} ({\vec {k}}_0 - \delta _k \hat{e}_l))/(2 \delta _k)$, respectively. Similar to the case of $H^{(1)}_{(\text {SO;1})}$, the off-diagonal terms in the sublattice degree of freedom in both $H^{(1)}_{(\text {SO;2})}({\vec {k}}_0)$ and $\partial _{k_l} H^{(1)}_{(\text {SO};2)} ({\vec {k}}_0)$ turn out to be either zero or negligibly small, and are dropped from further consideration. In addition, for both matrices, the diagonal terms corresponding to the two sublattices also turn out to have equal magnitudes but opposite signs. Hence, we can express the linear expansion in Eq. (19) as

20 $$\begin{aligned} H^{(1)}_{(\text {SO;2})}({\vec {k}}_0 + \delta {\vec {k}} ) \approx \sum _{l\in \{x,y\}} \sum _{s\in (x,y,z)} (h_{s;2}+h^{l,s}\delta k_l)\sigma _s t_z \end{aligned}$$

where the coefficients in the above expansion are obtained as

21 $$\begin{aligned} h_{s;2}&= {\frac{1}{4}}{\text {Tr}} \left( \tau _z\sigma _s H^{(1)}_{({\text{SO}};2)}({\vec {k}}_0)\right) , \end{aligned}$$

22 $$\begin{aligned} h^{l,s}&= {\frac{1}{4}} {\text {Tr}} \left( \tau _z\sigma _s \partial _{k_l}H^{(1)}_{({\text{SO}};2)}({\vec {k}}_0) \right) . \end{aligned}$$

Both the first and second-order SOI terms are diagonal in the sublattice index, implying that they comprise of either *A*–*A* or *B*–*B* couplings that correspond to the second nearest-neighbour couplings. Similar to the form of the second nearest-neighbour coupling terms in the case of the spinless Hamiltonian of Eq. (8), the effective single-orbital SOI Hamiltonian $H^{(1)}_{{\text{SO}}}$ would take the form of

23 $$\begin{aligned} H^{(1)}_{{\text{SO}}}({\vec {k}})&= \sum _{s \in \{x,y,z\}} i\left( t^s_{21} \text {e}^{i{\vec {k}}\cdot (\vec {a}_1-\vec {a}_2)} +t^s_{31}\text {e}^{i{\vec {k}}\cdot \vec {a}_1}+t^s_{32}\text {e}^{i{\vec {k}}\cdot \vec {a}_2}\right) \tau _z\sigma _s + \text {h.c.} \end{aligned}$$

We determine the coefficients $t^{s}_{mn}$ in the above, which represent the spin-dependent second nearest-neighbour couplings, by linearly expanding equation (23) around ${\vec {k}}_0$, and matching the terms in the expansion with those of Eqs. (17) and (19). Explicitly, the expansion of Eq. (23) is given by

24 $$\begin{aligned} H^{(1)}_{{\text{SO}}}({\vec {k}}+\delta {\vec {k}}) = H^{(1)}_{{\text{SO}}}({\vec {k}}_0) +\partial _{{\vec {k}}} H^{(1)}_{{\text{SO}}}({\vec {k}})\cdot \delta {\vec {k}} , \end{aligned}$$

where

25 $$\begin{aligned} H^{(1)}_{\text {SO}}({\vec {k}}_0)&= -\sum _{s\in \{x,y,z\}}2\tau _z\sigma _s ( i_1 t^s_{31} + i_2 t^s_{32} + i_{12} t^s_{21}), \end{aligned}$$

26 $$\begin{aligned} \partial _{{\vec {k}}} H^{(1)}_{{\text{SO}}}({\vec {k}})&= -\sum _{s\in \{x,y,z\}} 2\tau _z\sigma _s \left( (r_1 t^s_{31} + r_{12}t^s_{21})\vec {a}_1 + (r_2t^s_{32}-r_{12}t^s_{21})\vec {a}_2\right) . \end{aligned}$$

By term-wise comparison between the above expansion and their counterparts in Eqs. (20) to (22), the single-orbital TB coefficients related to the SOI interaction are given by

27 $$\begin{aligned} \begin{pmatrix} t^s_{31} \\ t^s_{32} \\ t^s_{21} \end{pmatrix} = -2 \begin{pmatrix} r_1 a_{1x} &{} r_2 a_{2x} &{} r_{12} (a_{1x}-a_{2x}) \\ r_1 a_{1y} &{} r_2 a_{2y} &{} r_{12} (a_{1y}-a_{2y}) \\ i_1 &{} i_2 &{} i_{12} \end{pmatrix}^{-1} \begin{pmatrix} h^{xs} \\ h^{ys} \\ h_{s;2} + h_{s;1}\end{pmatrix} . \end{aligned}$$

for each spin component $s \in (x,y,z)$. There is an additional spin and pseudo-spin independent energy shift $\delta E_0$ in $H^{(1)}_{(\text {SO};2)}$ given by $\delta E_0 = {\text {Tr}}(H^{(1)}_{(\text {SO};2)})/4$. Combining both the spinless and SOI components, the full single-orbital Hamiltonian with SOI takes the form of

28 $$\begin{aligned} H^{(1)S}&= H^{(1)}\otimes {\mathbf {I}}_\sigma +H_{\text {SO}}^{(1)}=((E_0 + \delta E_0) {\mathbf {I}}_\tau + t_3 \tau _x) {\mathbf {I}}_\sigma \nonumber \\+ & {} \sum _{i\in \{1,2\}} \left( \left( (t_{3i}{\mathbf {I}}_\tau + t_i \tau ^+){\mathbf {I}}_\sigma + \sum _{s\in \{x,y,z\}} t^s_{3i} \tau _z\sigma _s \right) \exp (i{\vec {k}}\cdot \vec {a}_i) \right) \nonumber \\+ & {} \left( \left( t_{21} {\mathbf {I}}_\tau {\mathbf {I}}_\sigma + \sum _{ s\in \{x,y,z\}} t^s_{21}\tau _z\sigma _s\right) \exp (i{\vec {k}}\cdot (\vec {a}_1-\vec {a}_2)) \right) + \text {h.c.} \end{aligned}$$

In the above, $$\tau ^+ \equiv \begin{pmatrix} 0 &{} 1 \\ 0 &{} 0 \end{pmatrix}$$, $t_1$ and $t_2$ are given by equation (10), $t_3$ by equation (11), $t_{31}$, $t_{32}$ and $t_{21}$ by equation (14), and the $t^s_{21}$, $t^s_{31}$, $t^s_{32}$ by Eq. (27).

Most studies on silicene and related materials would focus on electron energies close to the Dirac points. Hence, it would be useful to obtain the low-energy expansion of Eq. (28) around a Dirac point. This takes the general form of

29 $$\begin{aligned} H(\delta {\vec {k}})&= \sum _{j = x,y} \left( \nu (v_{jx}\tau _x + v_{j0}{\mathbf {I}}_\tau ) + v_{jy}\tau _y\right) \delta k{\mathbf {I}}_\sigma + \nonumber \\&\sum _{\beta =(x,y,z)} \left( \nu s_{0\beta } +\sum _{j=x,y} s_{j\beta }\delta k_j\right) \tau _z\sigma _\beta + (E_0+\delta E_0) {\mathbf {I}}_\tau {\mathbf {I}}_\sigma . \end{aligned}$$

In Eq. (29), we adopt the convention that $v_{j\alpha }$ are the coefficients of $\delta k_j\tau _\alpha {\mathbf {I}}_\sigma$ for $\alpha \in \{0,x,y,z\}$, and $s_{j\beta }$ the coefficients of $\delta k_j\tau _z\sigma _\beta$ for $j\in \{x,y,z\}$. $\nu =\pm 1$ denotes the valley index corresponding to the two Dirac points in the Brillouin zone. The coefficients $v_{j\alpha }$, $s_{i\beta }$ can be calculated from equation (28) via

30 $$\begin{aligned} v_{j\alpha } = {\frac{1}{4}} {\text {Tr}}(\partial _{k_j} H^{(1)S}\tau _\alpha ) \left| _{{\vec {k}}={\vec {k}}_0}, s_{i\beta } = {\frac{1}{4}} {\text {Tr}} ( \partial _{k_j} H^{(1)S}\tau _z\sigma _\beta ) \right| _{{\vec {k}}={\vec {k}}_0}. \end{aligned}$$

## Hamiltonian of strained silicene

In the previous section, we have derived the single-orbital tight-binding Hamiltonian that is applicable for any silicene lattice with arbitrary bond vectors ${\vec {d}}_1$, ${\vec {d}}_2$ and ${\vec {d}}_3$. We now consider a silicene lattice subject to the specific strain configuration of a uniaxial strain applied at an angle $\alpha$ to the *x*-axis. This has the effect of elongating lattice dimensions parallel to strain direction $\alpha$ by a factor $\gamma$ (say), and compressing the dimensions in the in-plane and out-of-plane directions perpendicular to $\alpha$ by $\eta (\gamma -1)$ and $\eta _\perp (\gamma -1)$ times respectively where $\eta$ and $\eta _\perp$ are the in-plane and out-of-plane Poisson ratios. Explicitly, the bond vectors ${\vec {d}}_i$ under the above strain configuration are given by

31 $$\begin{aligned} {\vec {d}}_i = {\mathbf {M}}^{-1} \begin{pmatrix} \gamma &{} 0 &{} 0 \\ 0 &{} 1 - \eta (\gamma -1) &{} 0 \\ 0 &{} 0 &{} 1-\eta _\perp (\gamma -1) \end{pmatrix} {\mathbf {M}}{\vec {d}}^{(0)}_i, \end{aligned}$$

where

$$\begin{aligned} {\mathbf {M}} \equiv \begin{pmatrix} \cos \alpha &{} -\sin \alpha &{} 0 \\ \sin \alpha &{} \cos \alpha &{} 0 \\ 0 &{} 0 &{} 1 \end{pmatrix} \end{aligned}$$

is the rotation matrix, and ${\vec {d}}^{(0)}_i$s are the unstrained bond vectors, which are given by ${\vec {d}}^{(0)}_1 = (1.930,1.114,-0.462)\ \text {\AA }$, ${\vec {d}}^{(0)}_2 = (-1.930,1.114,-0.462)\ \text {\AA }$, and ${\vec {d}}^{(0)}_3 = (0, -2.229, -0.462)\ \text {\AA }$. The strained bond vectors ${\vec {d}}_i$s are then substituted into Eqs. (1) and (2) in Section IIA to obtain the Hamiltonian for the strained silicene system.

**Figure 2:**
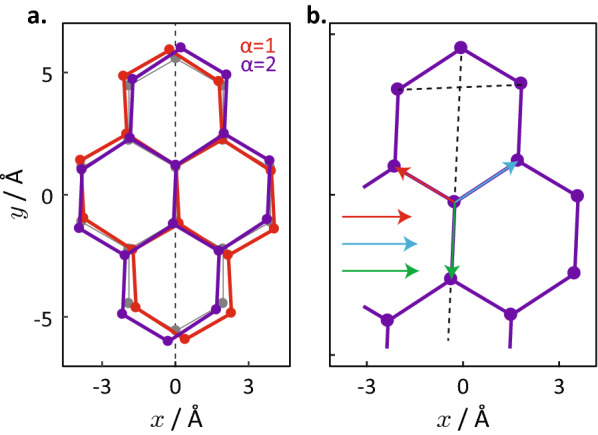
Compraison between strained and unstrained silicene lattices. (**a**) The unstrained silicene lattice (gray) and strained silicene lattices subject to uniaxial strain in the directions of $\alpha =1$ rad (red) and $\alpha =2$ rad (blue) with strain magnitude of $\gamma =1.1$ (i.e. 10% elongation) for both. (**b**) A zoomed-in view of the strained silicene lattice for $\alpha =2$ rad and $\gamma =1.1$. The black dotted lines illustrate the breaking of the reflection symmetry under strain—the lines do not cross at right angles to each other. The three coloured arrows represent the displacement vectors ${\vec {d}}_1$, ${\vec {d}}_2$ and ${\vec {d}}_3$ from the central lattice point to its nearest neighbours. The arrows are redrawn and arranged horizontally at the lower left corner for comparison of their relative lengths.

Figure 2 illustrates the strained silicene crystal lattice for two different strain directions of $\alpha =1$ and 2 rad. A strain along $\alpha < \pi /2$ (illustrated by $\alpha = 1$ rad of Fig. 2a) tilts the lattice in the counter-clockwise direction, while strain along $\alpha > \pi /2$ (illustrated by $\alpha =2$ in Fig. 2) tilts the lattice in the clockwise direction.

The introduction of strain along a skew direction that differs from the symmetry axes along $\alpha =0$ and $\alpha =\pi$ has two effects: (1) It breaks the mirror symmetries of the lattice. While it is obvious that the reflection symmetries along the *x* and *y* axes are broken, the same also occurs to the axes of reflection symmetry along the armchair and zigzag directions. Figure 2b illustrates the breaking of these reflection symmetries—the armchair and zigzag directions are not exactly perpendicular to each other in the distorted lattice. (2) The skew strain also breaks the isotropy of the displacement between a given lattice site to its three nearest neighbours. In unstrained silicene, the separation distances between a lattice site to its three nearest neighbours are equal and the angular displacement between each neighbour is $2\pi /3$, both of which no longer hold in strained silicene.

**Figure 3:**
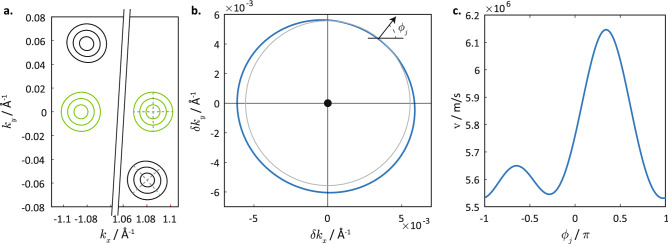
Equal enegy-contours and group velocities of silicene. (**a**) The EECs for strained (black) silicene for strain direction $\alpha =2$ and magnitude $\gamma =1.1$, and unstrained silicene (green). The three contour lines correspond to energies of 20 meV, 40 meV and 60 meV above their respective Dirac points. The grey dotted lines straddling the positive $k_x$ EECs denote the major and minor axes of the EECs. (**b**) A close-up of the EEC of strained silicene in the vicinity of the positive $k_x$ valley (blue line) at $60\ \text {meV}$ above the Dirac point. The EEC lies off-centre about the Dirac point. The thinner gray circle is of uniform radius and centered at the Dirac point to illustrate the anisotropy of the EEC. The angle $\phi _j$ (depicted at the upper right of the EEC) denotes the direction of the normal to the EEC, which is also the velocity direction corresponding to the state at the *k* point. (**c**) Plot of the velocity as a function of the velocity direction $\phi _j$ taken at each *k*-point on the EEC of strained silicene in (**b**).

The breaking of the reflection symmetries as well as of the nearest-neighbour isotropy are reflected in the bandstructure of the strained silicene. Figure 3 illustrates the Equal Energy Contours (EECs) at small energies above the respective Dirac points for both strained and unstrained silicene. Note that ${\vec {k}}$ denotes the electron wavevector over the entire Brillouin zone, while $\delta {\vec {k}}$ denotes the small displacement of the wavevector from its respective Dirac point. Let us first consider the EECs of *unstrained* silicene. The states in the vicinity of the Dirac points are distributed over two valleys, each centered about $k_y=0$ and separated along the $k_x$ axis (Fig. 3a). This separation of the states into two valleys along the $k_x$ direction can be explained by reflection and time-reversal symmetries, and our choice of the unit cell and coordinate system of the lattice, as shown in Fig. 1.

Let us consider the effect of reflection symmetry on the Hamiltonian and bandstructure of silicene. In unstrained silicene, there is reflection symmetry about the *y*-axis. A reflection about the *y*-axis leads to the replacement of $k_y \rightarrow -k_y$. In addition, it also swaps the *A* and *B* sublattice sites with one another, which brings about the following replacements of the pseudospin terms: $\tau _y \rightarrow -\tau _y$ and $\tau _z \rightarrow -\tau _z$. Thus, the only terms linear in $\delta {\vec {k}}$ that are allowed by reflection symmetry about the *y*-axis are $\delta k_y \tau _y$, $\delta k_y \tau _z$, $\delta k_x\tau _x$ and $\delta k_x{\mathbf {I}}$. At the same time, the silicene system also obeys time-reversal symmetry. Time-reversal is effected by (1) the replacement of $\delta {\vec {k}}\rightarrow -\delta {\vec {k}}$ and (2) considering a spinless system for now, taking the complex conjugate, which results in $\tau _y \rightarrow -\tau _y$ (n.b. $\tau _x$ and $\tau _z$ are unaffected because all their elements are real. We will discuss the spin-ful system in the next paragraph). Suppose the Dirac point were to lie at the origin $(k_x,k_y)=(0,0)$. Upon linear expansion in $\delta k_x$ and $\delta k_y$, the only terms allowed by time-reversal symmetry are $\delta k_x\tau _y$ and $\delta k_y \tau _y$. The latter term is consistent with *y*-reflection symmetry, as well as the *x*-reflection symmetry which is present in the system. The former term however violates both the *x* and *y*-axis reflection symmetries. The only way out that preserves both the time-reversal and reflection symmetries is to have reflection-symmetric pairs of states separated by a finite separation in *k*-space along the $k_x$ axis. These considerations lead to the existence of two valleys, as evident in the EECs of Fig. 3a, where the time-reversal and *x*-reflection partner of a state in one valley lies in the other valley. They also explain the emergence of the $\nu$ chirality factor in the spinless single-orbital low-energy linear expansion for silicene:

32 $$\begin{aligned} H = v_f( \delta k_y \tau _y + \nu \delta k_x\tau _x) \end{aligned}$$

appearing as part of the Hamiltonian in many theoretical works on unstrained silicene (e.g. Ref.^15,53^)—the factor of $\nu$ comes about because for a state that is displaced by $\delta k_x$ with respect to the Dirac point at one valley, its counterpart at the other valley would be displaced by $-\delta k_x$ with respect to its own Dirac point. Note that in unstrained silicene, the velocity terms in our low-energy Hamiltonian of Eq. (29) would be given by $v_{j(x,y)} = v_f\delta _{j,(x,y)}$ and $v_{j0}=0$, so that the Hamiltonian would reduce to Eq. (32) (discounting the spin degree of freedom).

Let us now consider the symmetries involved in the low-energy single-orbital Hamiltonian with spin, as described by Eq. (29). The valley index $\nu$ appears together with the spin-independent terms containing $\tau _x$ and the $\delta {\vec {k}}$-independent spin terms because of time-reversal symmetry. This is because time-reversal flips the signs of $\delta {\vec {k}}$, $\tau _y$, ${\vec {\sigma }}$ and $\nu$ (as mentioned above, the time-reversal partner of a state in a valley at $\delta {\vec {k}}$ in one valley is located at $-\delta {\vec {k}}$ in the other valley). Thus to preserve the time reversal symmetry, it can be seen that every term in Eq. (29) consists of an even number of terms from the set comprising $\{\delta {\vec {k}},\tau _y,\nu ,{\vec {\sigma }}\}$.

We now consider the effects of applied strain on the EECs and the Dirac points. A small strain would only perturb the dispersion relation slightly, so that the basic dispersion structure of two valleys separated largely along the $k_x$ direction still holds. The approximate (but not exact) reflection symmetry of the strained silicene lattice along the strained armchair direction is mirrored by the approximate reflection symmetry of the EECs and Dirac points of the two valleys, as shown in Fig. 3a. (The SOI splitting of the energy bands is not evident to the naked eye at the scale of the figure). Note that although the exact reflection symmetry is broken in strained silicene, time-reversal symmetry is still preserved. Thus, for any state with energy *E* at ${\vec {k}}$, there still exists a corresponding counterpart state with the same energy at $-{\vec {k}}$.

The application of strain would also introduce an anisotropy to the EEC as shown in Fig. 3b, which plots the EEC of strained silicene for one particular valley and energy. In unstrained silicene, the low-energy EEC is circular about the Dirac point, reflecting the isotropy of the system. However, in strained silicene, the EEC has a non-uniform radius, which may be ascribed to the anisotropy of the velocity around the EEC, as plotted in Fig. 3c. Recall that the velocity is defined as $\vec {v} = \langle \nabla _{{\vec {k}}} \hat{H} \rangle$. This implies that: (1) the direction of the velocity follows that of the outward normal on the EEC, and (2) the change in the wavevector $\delta k$ per unit change in energy at a given point on the EEC is inversely proportional to the velocity there. In other words, $\delta {\vec {k}} = \int (\partial k/\partial E)\ \text {d}E = \int 1/v\ \text {d}E$. Therefore, points on the EEC with greater electron velocity would have $\delta {\vec {k}}$ of smaller magnitude, and lie closer to the Dirac point, giving rise to the non-uniform radius of the EEC. The anisotropy in the velocities, shown in Fig. 3c, can be traced to the anisotropy in the bond lengths of a lattice site to its three nearest neighbours in the strained silicene lattice. A smaller spatial separation between neighbouring sites would lead to a larger hopping parameter between these sites and thus larger carrier velocity.

**Figure 4:**
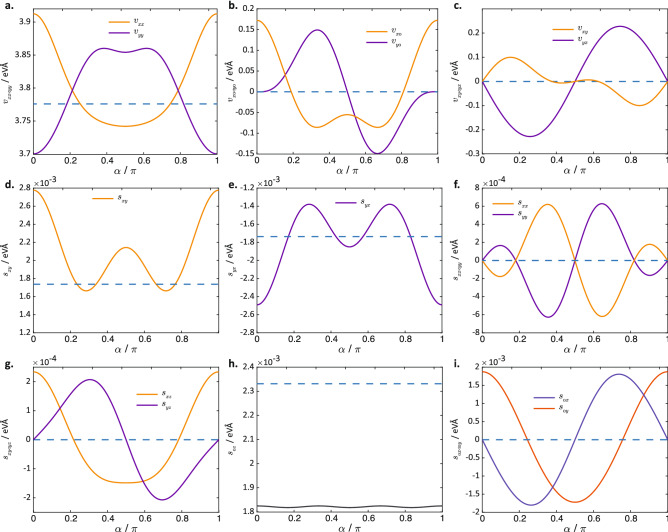
The values of the various Hamiltonian parameters for strained silicene with $\gamma = 1.1$ as a function of the strain angle $\alpha$ in Eq. (29) where for $j\in (x,y),\bar{j}\ne j$. The dotted lines in each plot shows the values of the quantities plotted in unstrained silicene. (**a**) $v_{jj}$, (**b**) $v_{ji}$, (**c**) $v_{j\bar{i}}$, (**d**) $s_{xy}$, (**e**) $s_{yx}$, (**f**) $s_{jj}$, (**g**) $s_{jz}$, (**h**) $s_{0z}$, (**i**) $s_{0j}$.

We now consider the effect of varying the strain direction, i.e. angle $\alpha$, on the various coefficients of the low-energy Hamiltonian in Eq. (29), while keeping the strain magnitude fixed at $\gamma =1.1$ (see Fig. 4). We start with the special cases of strain along $\alpha =0$ and $\alpha =\pi /2$, which preserve the *x* and *y*-reflection symmetries of the silicene lattice. The major and minor axes of the EEC ellipses at $\alpha =0,\pi /2$ lie parallel to the $k_x$ and $k_y$ axes, in agreement with the *x* and *y*-reflection symmetries of the crystal axes. The strain causes the velocities $v_{xx}$ and $v_{yy}$ to differ from each other (Fig. 4a), so that the EECs are no longer perfect circles. The strain also results in a finite $v_{x0}$ and zero $v_{y0}$ (Fig. 4b). At $\alpha =0$, the finite $v_{x0}$ term tilts the Dirac cone along the $k_x$ direction, so that the EEC assumes the shape of a tilted ellipse and the projection of the Dirac point no longer lies at the geometric centre of the EEC. Conversely, when strain is applied along $\alpha =\pi /2$ , we have finite $v_{y0}$ and zero $v_{x0}$, which translates to a tilt of the Dirac cone in the $k_y$ direction. A strain which deviates away from the special angles of $\alpha =0$ and $\alpha =\pi /2$ leads to finite values of $v_{y0}$ (Fig. 4b) and $v_{l\bar{l}}$ where $\bar{l} = x (y)$ for $l = y (x)$ (Fig. 4c). $v_{l\bar{l}}$ and $v_{y0}$ have the effect of rotating the EECs in *k*-space so that the major and minor axes of the EEC ellipses no longer lie along the *x* and *y*-axes but are now oriented approximately along the direction of the reflection axes of the distorted crystal lattice. An example of a rotated EEC in strained silicene is plotted in Fig. 3a in which the axes of the EECs are denoted by the dotted lines. (We also plot the energies and *k*-space positions of the Dirac points for strained silicene at different strain angles in the “Suppementary Note”.)

Next, we now consider the spin-dependent terms shown in Fig. 4d–i. In unstrained silicene, the SOI terms near the Dirac points take the form of

33 $$\begin{aligned} \lambda _{\text {SO}}\sigma _z + \lambda _{\text {R}}(\delta {\vec {k}}\times \hat{z})\cdot {\vec {\sigma }} \end{aligned}$$

in the notation of Ref.^13^ where $\lambda _{\text {SO}}$ is the strength of a $\delta {\vec {k}}$-independent SOI component pointing in the out-of-plane direction, and $\lambda _{\text {R}}$ is the strength of the SOI term with a Rashba-like symmetry. This corresponds to $s_{j\beta }$ in Eq. (29) taking the form of $s_{j\beta } = \lambda _{R}\epsilon _{j,\beta , z} + \lambda _{\text {SO}}\delta _{\beta ,z}$, as can be seen from a term-wise comparison of the coefficients of $\sigma _\beta$ and $\delta k_j\sigma _\beta$. The application of strain along the special directions of $\alpha =0$ and $\pi /2$ leads to $s_{xy} \ne -s_{yx}$ (see Fig. 4d,e). The breaking of the anti-symmetry between $s_{xy}$ and $s_{yx}$ means that the direction of the in-plane SOI field, which is given by $(s_{yx}\delta k_y,s_{xy}\delta k_x, 0)$ is no longer perpendicular to $\delta {\vec {k}}$ when $\delta k_x \ne 0$ or $\delta k_y \ne 0$, and differs from the simple Rashba form of $s_{yx}(k_y,-k_x, 0)^T\cdot {\vec {\sigma }}$. These effects persist for other values of $\alpha$ besides $\alpha =0,\pi /2$. For an arbitrary strain angle, the breaking of the three-fold rotational and reflection symmetries results in the emergence of additional SOI terms that are absent in unstrained silicene: (1) Strain coefficients $s_{jj}$ where $j\in \{x,y\}$ (see Fig. 4f) due to the breaking of the *x* and *y*-reflection symmetries. These give rise to spins that are parallel to $\delta {\vec {k}}$, and thus lead to in-plane spins deviating even further from the direction perpendicular to ${\vec {k}}$, in addition to the effect of $s_{xy}\ne s_{yx}$. (2) Strain coefficients involving $s_{xz}$ and $s_{yz}$ (see Fig. 4g) due to breaking of the rotational symmetry. These give rise to out-of-plane spin, i.e., $\sigma _z$, which are ${\vec {k}}$-dependent, and hence can be induced or modulated by current. (3) Strain coefficients $s_{0x}$ and $s_{0y}$ (see Fig. 4i). These are $\delta {\vec {k}}$-independent terms associated with in-plane spins. Note that in the absence of strain, the only $\delta {\vec {k}}$-independent terms are those associated with the out-of-plane spins (see Fig. 4h—n.b. $s_{0z}$ is already non-zero in the absence of strain, and the application of strain merely changes its magnitude). To summarize, applying strain removes the constraints imposed by rotational and reflection symmetries, and induces the emergence of $\delta {\vec {k}}$-dependent and $\delta {\vec {k}}$-independent terms with spin components in all three directions. In the next section, we illustrate the consequence of these additional SOI terms in strained silicene and their possible role in spin torque applications.

## Current-induced spin accumulation

As an exemplary application of our derived Hamiltonian, we study the out-of-plane spin accumulation in strained silicene induced by an in-plane current or electric field ${\mathcal {E}}_y$ in the *y*-direction. The silicene system is additionally coupled to an in-plane magnetization $M_x$ along the *x*-direction, and an out-of-plane electric field ${\mathcal {E}}_z$. This ${\mathcal {E}}_z$ field may, for instance, be induced by an out-of-plane gate voltage^14^ or from substrate effects^54^, as a result of the *A* and *B* sublattices being affected differently by the substrate due to the lattice buckling. The low-energy Hamiltonian $H'$ thus takes the form of

34 $$\begin{aligned} H'(\delta {\vec {k}}) = H(\delta {\vec {k}}) + M_x\sigma _x + {\mathcal {E}}_z\tau _z \end{aligned}$$

where *H* is given by Eq. (29). An in-plane magnetization coupling will bring out the effect of two particular terms in the strained silicene Hamiltonian that have not been studied previously: the $\delta {\vec {k}}$-independent in-plane SOI field (Fig. 4i) and the $\delta {\vec {k}}$-dependent out-of-plane SOI field (Fig. 4g). As we shall show later, the first term allows an *in-plane* magnetization to lift the energy degeneracy between the two valleys, while the combination of the two terms breaks the *k*-space antisymmetry of the current-induced spin-*z* accumulation. This results in a net out-of-plane spin accumulation that is crucial for spin–orbit torque magnetization switching in silicene-based systems^55–57^.

In the relaxation time approximation, the electric field ${\mathcal {E}}_y$ shifts the Fermi surface by an amount $\Delta k_y = \tau _R {\mathcal {E}}_y$ where $\tau _R$ is the relaxation time. The resulting change in the field-induced accumulation value of an observable quantity *O* per unit area of the silicene is then given by

35 $$\begin{aligned} \Delta O&= \left( \sum _b \int _{S_b} d{\vec {k}}~g_b({\vec {k}})v_{y,b}({\vec {k}})\langle {\hat{O}}({\vec {k}},b)\rangle \right) \Delta k_y\equiv (\delta O)\Delta k_y \end{aligned}$$

where the integration in *k*-space is over the Fermi surface $S_b$, and the summation *b* is over the occupied bands (the subscript *b* refers to the band index and the integral $\int _{S_b} d{\vec {k}}$ is a line integral over the one-dimensional Fermi surfaces of the two-dimensional system). Here, $g_b({\vec {k}}) = {\frac{1}{(2\pi )^3 |v_b({\vec {k}})|}}$ is the density of states of the band, $v_{y,b}({\vec {k}})$ is the velocity in the *y*-direction, and $\langle {\hat{O}}({\vec {k}},b) \rangle$ is the expectation value of the operator ${\hat{O}}$ for band *b* at ${\vec {k}}$. $\delta O$ is the change in the accumulation value of the quantity *O* per unit $\Delta k_y$ per unit area after *k*-space integration over the Fermi surfaces of all the occupied bands and summation over the bands. $\delta O$ hence has the dimensions of *O* multiplied by an inverse length unit.

**Figure 5:**
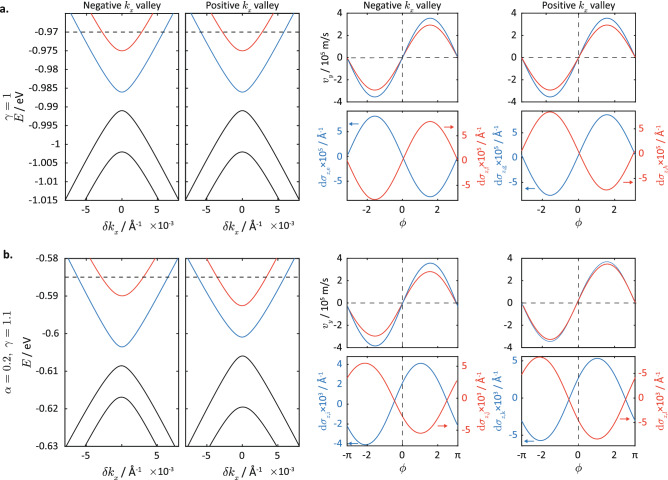
Comparison of the bandstructure and current-induced spin accumulation between strained and unstrained silicene. (**a**)The dispersion relations at $\delta k_y=0$, expectation values of the group velocity $v_y$ and out-of-plane spin accumulation offsets $\text {d}\sigma _{z,a} \equiv \langle \sigma _z\rangle - \sigma ^0_a$, $a = (\text {e}, \text {f},\ldots,\text {l})$ at the positive $k_x$ (left) and negative $k_x$ (right) valleys for unstrained silicene at electron energy $E=-0.97$ eV. (**b**) The corresponding plot to (a) strained silicene with $\alpha =2,\gamma =1.1$ at $E=-0.585\ \text {eV}$, with an applied $M_x=5$ meV and $E_z = 8$ meV. Here, $\sigma ^0_{\text {e}}=- \sigma ^0_{\text {g}}=-0.15708\ \text {\AA }^{-1}$, $\sigma ^0_{\text {f}}=- \sigma ^0_{\text {h}}=0.27132\ \text {\AA }^{-1}$,$\sigma ^0_{\text {i}}=-0.110\ \text {\AA }^{-1}$,$\sigma ^0_{\text {j}}=-0.183\ \text {\AA }^{-1}$, $\sigma ^0_{\text {k}}=0.125\ \text {\AA }^{-1}$, and $\sigma ^0_{\text {i}}=-0.212\ \text {\AA }^{-1}$. The electron energies of $E=-0.97$ eV and $E=-0.585$ eV are indicated by dotted lines in the dispersion relation plots in (**a**) and (**b**), respectively. The colours of the $\langle \partial _{k_y} H \rangle$ and $\langle \sigma _z\rangle$ plots correspond to the bands in the dispersion relations plotted with the respective colours.

We consider the specific case of ${\hat{O}}=\sigma _z$ corresponding to the out-of-plane spin accumulation. We therefore first study how strain affects the bandstructure, as well as the expectation values $\langle \sigma _z ({\vec {k}},b) \rangle \equiv \langle {\vec {k}},b|\sigma _z|{\vec {k}},b\rangle$ and $v_{y,b} = \langle {\vec {k}},b| \partial _{k_y} H |{\vec {k}},b \rangle$ that are needed for calculating $\Delta \sigma _z$ using equation (35) . Figure 5 plots the dispersion relations, and the expectation values of $v_{y}$ and $\sigma _z$ in unstrained silicene, as well as in silicene under strain of direction $\alpha =2$ rad, and magnitude $\gamma =1.1$, calculated using the low-energy Hamiltonian Eq. (34). Here, we assume $M_x=5\ \text {meV}$ and ${\mathcal {E}}_z=8\ \text {meV}$. The expectation values are plotted for the two conduction-like bands corresponding to a specific energy value of $E=-0.585$ eV ($E=-0.97$ eV) for strained (unstrained) silicene, as functions of the polar angle $\phi$ of the EEC, defined as $\phi \equiv \tan ^{-1} (\delta k_y / \delta k_x)$.

In unstrained silicene, the dispersion relation exhibits a valley symmetry such that the energy at a given $\delta {\vec {k}}$ at one valley is equal to the energy at $-\delta {\vec {k}}$ at the other valley (Fig. 5a). This degeneracy is lifted in the dispersion relation for strained silicene (Fig. 5b). This lifting of the degeneracy is due to the finite $\delta {\vec {k}}$-independent spin coefficient $s_{0x}\sigma _x$ in strained silicene, which lies parallel to the applied magnetization. We can see this clearly by the fact that the Hamiltonian at the Dirac point $\delta {\vec {k}}=0$ in the presence of an applied magnetization ${\vec {m}}_a$ (the subscript ‘a’ denoting ‘applied’) and out-of-plane electric field ${\mathcal {E}}_z$ takes the form of

36 $$\begin{aligned} H'(\delta {\vec {k}}=0) = E_0 + ({\vec {m}}_a + \nu \vec {S}_0\tau _z)\cdot \sigma + {\mathcal {E}}_z\tau _z \end{aligned}$$

where $E_0$ is the constant shift in energy, $\nu = \pm 1$ denotes the valley index and $\vec {S}_0 \equiv (s_{0x},s_{0y},s_{0z})$ is the net SOI field at the Dirac point (see Eq. (29)). Equation (36) is a $(4\times 4)$ matrix due to the presence of both the A/B sublattice ($\tau$) and real spin ($\sigma$) degrees of freedom. There are, correspondingly, four eigenvalues at each valley labeled by the four possible combinations of $\pm _{(\tau )}$ and $\pm _{(\sigma )}$ given by

37 $$\begin{aligned} E_{\pm _{(\tau )},\pm _{(\sigma )}} = \pm _{(\sigma )} | {\vec {m}}_a \pm _{(\tau )}\nu \vec {S}_0 | \pm _{(\tau )} {\mathcal {E}}_z. \end{aligned}$$

where $\nu$ is the valley index in Eq. 29.

We first consider the scenario where $\vec {S}_0$ is perpendicular to ${\vec {m}}_a$. This corresponds to the case of unstrained silicene in Fig. 5a where $\vec {S}_0=\lambda _{\text {SO}}\hat{e}_z$ lies on the *z*-direction (see Eq. (33)) and ${\vec {m}}_a$ in the *x* direction. In this case, the $| {\vec {m}}_a \pm _{(\tau )}\nu \vec {S}_0) |$ term in Eq. (37) reduces to $\sqrt{ |\vec {S}_0|^2 + |{\vec {m}}_a|^2 }$, which has no valley dependence. Despite the breaking of time-reversal symmetry by ${\vec {m}}_a$, for every state with energy *E* at a $\delta {\vec {k}}$ in one valley, there exists a corresponding state with the same energy at $-\delta {\vec {k}}$ in the other valley. (Note that this pair of degenerate states are not time-reversal partners of each other because the expectation values of the real spin along the direction of the applied magnetization for both states have the same signs.)

The scenario where ${\vec {m}}_0$ has components parallel to ${\vec {m}}_a$ is more interesting. This occurs for the strained silicene in Fig. 5b where $\vec {S}_0$ has a finite *x* component parallel to ${\vec {m}}_a$, i.e., $s_{0x}$ (c.f. Fig. 4i). In this case, the coupling to the magnetization increases (decreases) the energy spacing between the hole (particle) states at one valley while effecting the reverse at the other valley, thus lifting the energy degeneracy between the two valleys.

We now analyze the effects of strain on the particle velocity along the applied electric field, i.e., $v_y\equiv \langle \partial _{k_y} H\rangle$, and its variation on the EEC. Note that for any electric-field induced accumulation arising from the Fermi surface shift, the expectation value of the corresponding observable is multiplied by the velocity term, as can be seen in Eq. (35). In unstrained silicene, the exact *y*-reflection symmetry results in the upper (lower) half of the EECs having positive (negative) values of $v_y$ for both of the particle bands in each valley (see the upper right plots of Fig. 5a) with the dividing line between the two signs of $v_y$ falling exactly on the $\phi =0$ axis. Under strain, this *y*-reflection symmetry is broken, with the result that the major axis of the tilted elliptical EEC lies off the $k_x$ axis. Hence, the dividing line between the two signs of $v_y$ is slightly offset from $\phi =0$. This is evident in the plots in Fig. 5b where the curves for $v_y$ do not intercept $\phi =0$ and $\phi =\pi$ exactly at $v_y=0$.

Let us now consider the expectation value $\langle \sigma _z\rangle$ and its variation along the EECs of unstrained silicene. In each valley, the velocity $v_y$ varies as $\cos \phi$ owing to the isotropy of the EEC. Likewise, the value of $\langle \sigma _z\rangle$ for each band also shows a $\cos \phi$ variation of small amplitude set against a much larger constant offset, as shown in Fig. 5a. At a given Fermi energy above the band gap, the particle band with the higher band minimum has a larger magnitude of $\langle \sigma _z \rangle$, as shown in the upper right plots of Fig. 5a. (This is because the value of $\langle \sigma _z \rangle$ scales with the ratio of the magnitude of the $\delta {\vec {k}}$-independent $\sigma _z$ term to the sum of the $\delta {\vec {k}}$ dependent in-plane SOI terms and the applied *x* magnetization in the Hamiltonian, i.e. $|s_{0z}/\sqrt{\sum _\beta (\sum _{j} s_{j\beta }\delta k_j + M_x\delta _{x,\beta })^2}|$ for $j,\beta =\{x,y\}$. The band with a higher band minimum would have smaller values of $\delta {\vec {k}}$ leading to a larger ratio). The sinusoidal variation of $\langle \sigma _z \rangle$ in both bands seems to suggest that it would integrate to zero, but this is not the case. The electric field (${\mathcal {E}}_y$)-induced spin accumulation is proportional to the product of $v_y$ and $\langle \sigma _z \rangle$. This product is not a pure sinusoidal function and thus yields a net spin-*z* accumulation in each band after integrating over the contributions of all the states. However, when considering the contributions of the two valleys, the current-induced spin *z* accumulation would still be exactly zero. This is because the reflection symmetries in unstrained silicene would result in the value of $\langle \sigma _z\rangle$ for a given value of $\phi$ having equal magnitudes but opposite signs for corresponding states in the two valleys (see lower right plots in Fig. 5a).

In contrast, the breaking of the reflection symmetries in strained silicene leads to two effects: (1) the energy degeneracy between the two valleys is lifted, and so the spin-*z* accumulation in the two valleys would have different magnitudes for a given value of $\phi$ and do not cancel each other even after adding the two valley contributions; (2) in each valley, there is a more pronounced asymmetry of the spin-*z* expectation value about $\phi =0$ (see lower right plots in Fig. 5b); (3) the variation in $\langle \sigma _z\rangle$ with $\phi$ has a much larger amplitude compared to the unstrained case. Effect (2) is a consequence of the anisotropy of the Fermi surface due to its deviation from a perfect circular shape (see Fig. 3b). Effect (3) results from the larger variation of the ratio of the out-of-plane spin-*z* terms to the in-plane spin terms of the Hamiltonian with the direction of $\delta {\vec {k}}$. The magnitude of the spin-*z* terms gains an extra dependence on the direction of $\delta {\vec {k}}$ via terms involving $s_{xz}$ and $s_{yz}$ (plotted in Fig. 4g). Further, the magnitude of the in-plane spin terms also gains an extra dependence on $\delta {\vec {k}}$ due to the variation of the magnitude $|\delta {\vec {k}}|$ with angle $\phi$ on the EEC, the non-zero values of $s_{xx}$, $s_{xy}$ , $s_{yx}$ and $s_{yy}$, and the inequality $s_{xy}\ne s_{yx}$ (see Figs. 3f,g), all as a result of the symmetry-breaking of the strained lattice. Consequently, these factors result in a sizable electric-field induced spin-*z* accumulation in strained silicene.

**Figure 6:**
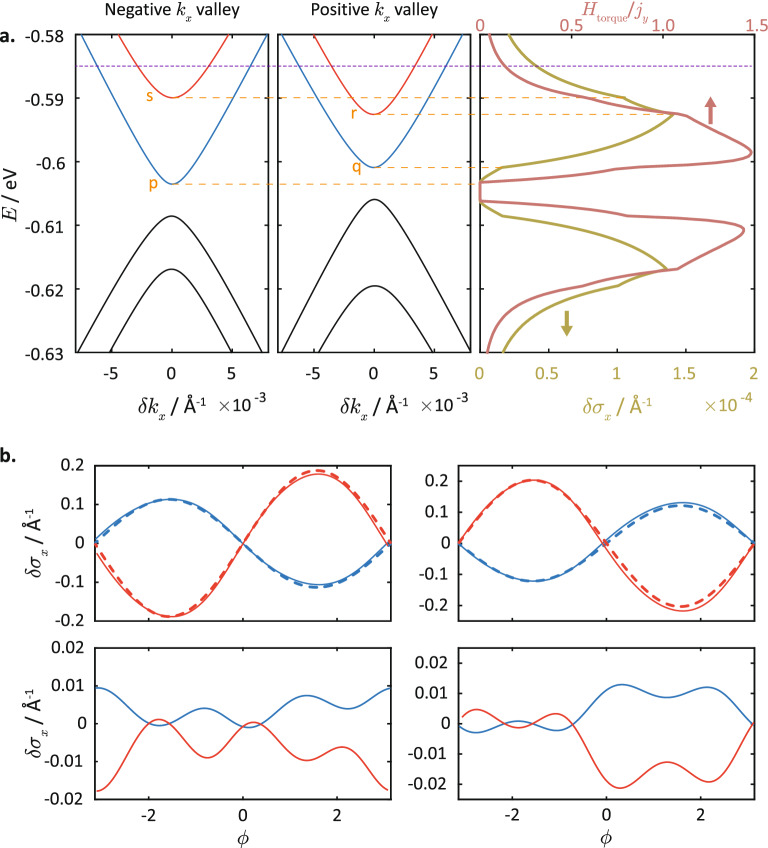
Bandstructure and spin accumulation in strained silicene. (**a**) The bandstructures at $\delta k_y=0$ around the Dirac point in (left) the negative $k_x$ valley and (center) positive $k_x$ valley in strained silicene, with strain magnitude $\gamma =1.1$ and strain direction $\alpha =2$, coupled to an in-plane magnetization $M_x=5$ meV, and electric field of $E_z= 8$ meV. The right plot shows the spin accumulation $\delta \sigma _z$, and the spin torque efficiency, i.e., the ratio of the effective magnetic field due to the torque exerted by the spin accumulation to the current density. (**b**) The solid lines in the top plots show the $\delta \sigma _z$ for the bands with the corresponding plot colours in (**a**), for electron energy $E=-0.585$ eV. For comparison, we plotted the sinusoidal $\sin \phi$ curves (dotted) to show the asymmetry of the $\delta \sigma _z$-dependence with variation in $\phi$. The bottom plots show $\delta \sigma _z$ after subtracting away off the antisymmetric $\sin \phi$ contribution, which clearly reveal the sign of the net spin accumulation.

Figure 6a shows the dispersion relations at both $k_x$ valleys, as well as the net spin accumulation $\delta \sigma _z$, as a function of the Fermi energy *E* for silicene under the same strain configuration as in Fig. 5. Starting from the minimum of the lowest energy particle band at the negative $k_x$ valley at $-0.6025$ eV (corresponding to level *p* in Fig. 6a), $\delta \sigma _z$ increases steadily with *E* until it reaches the minimum of the same band at the other (positive) $k_x$ valley corresponding to $E=-0.6009$ eV (level *q*). At this point, there is a kink due to $\delta \sigma _z$ increasing at a faster rate because the net $\delta \sigma _z$ contributions of the lower bands for *both* valleys are positive (refer to the blue curves in Fig. 6b). This trend continues until the Fermi energy hits the minimum of the higher energy particle band at the positive $k_x$ valley at $E=-0.5926$ eV (level *r*). $\delta \sigma _z$ then decreases from its maximum value because the net contribution from the higher energy particle band is negative. At $E=0.5899$ eV (level *s*), we reach the band minimum of the upper band at the other (negative) valley. Because the net contribution of this band is negative as well (see red curves in Fig. 6b), this translates into a greater rate of decrease in $\delta \sigma _z$ with increasing *E*, as shown by a kink in the $\delta \sigma _z$ variation in Fig. 6a. The net spin accumulation then approaches zero with further increase in energy, because the contributions for the two particle bands in each valley approach the same magnitude but are of opposite directions at large energies.

We now consider the effective magnetic field $H_{\text {torque}}$ due to the spin torque acting on the FM magnetization as a result of the out-of-plane spin accumulation. This is proportional to $\delta \sigma _z$ and the magnetization of the FM-doped silicene system (we assume a magnetic moment of $3 \mu _B$ per unit cell^58^). The spin torque efficiency is then given by the ratio of the effective torque field to the longitudinal current density $j_y$ (which has units of A/m in the 2D silicene system and is obtained by setting $O=e v_y$ in Eq. (35)). Both the torque field and the $j_y$ are proportional to the shift $\Delta k_y$ due to the electric field ${\mathcal {E}}_y$, and hence the torque efficiency is independent of ${\mathcal {E}}_y$. (The torque efficiency is dimensionless because both the current density and the effective magnetic field have dimensions of current divided by length.) In Fig. 6a, the torque efficiency is plotted as a function of the Fermi energy *E*. It exhibits a similar dependence on *E* as the spin accumulation $\delta \sigma _z$ with kinks at levels *p*, *q*, *r* and *s*, but the degree of modulation is enhanced as both $v_y$ and $\delta \sigma _z$ are varying with *E*. In addition, the torque efficiency reaches its peak value at a lower value of Fermi energy compared to that of $\delta \sigma _z$ peak, because of increasing $j_y$ between levels *q* and *r* (n.b. $j_y$ constitutes the denominator of the torque efficiency ratio). The large modulation of the torque efficiency with *E* indicates the possibility of optimizing it in strained silicene by fine-tuning the Fermi level.

We summarize the emergence of finite out-of-plane spin accumulation in the silicene system as being due to the interplay of three factors, i.e., strain, in-plane SOI field and magnetization. On its own, the in-plane magnetization cannot lift the energy degeneracy of the two valleys in unstrained silicene (as shown in Fig. 5a). The energy degeneracy between the two valleys in turn leads to the perfect cancellation of the current-induced accumulation $\delta \sigma _z$ due to the antisymmetry of $\delta \sigma _z$ in the two valleys. The situation changes in the presence of strain due to two factors: (1) the strain gives rise to in-plane components of the SOI field near the Dirac points, which break the energy degeneracy in the presence of an in-plane magnetization and (2) the strain leads to the breaking of the reflection symmetries in the silicene lattice. This results in a finite net $\sigma _z$ in each particle band (as shown in Fig. 6b). Moreover, the contributions to $\sigma _z$ of the corresponding bands in both valleys have the same signs. Hence, there is a out-of-plane spin accumulation $\delta \sigma _z$ in strained silicene after integration over the entire Fermi surface and summation over the valleys. This current-induced out-of-plane spin accumulation may potentially be applied to effect spin–orbit torque magnetization switching, similar to that demonstrated in magnetic layers coupled to materials with SOI, such as heavy metals and topological insulators^55–57,59–61^.

Although it may not be easy to realize actual applications such as the strain-induced spin torque switching described in the section at the moment because of the assumption of a homogenous ideal crystal structure and the need to apply the strain at a specific in-plane angle, the strain-induced spin accumulation described serves as an example of the possibilities that applying strain away from the high-symmetry armchair and zigzag directions may open. Such strains can possibly be applied by two recently reported techniques. In the first technique, the 2D material $\hbox {MoS}_2$ was grown on a stretchable substrate^62^. In the second technique, a pre-shaped HBN layer was added to a bilayer graphene layer to serve as a handle that can be pushed by an AFM tip to achieve highly controlled twist angles on the bilayer graphene^63^. This technique can be modified to realize strains at arbitrary desired angles.

## Conclusion

In this work, we derived a single-orbital tight-binding Hamiltonian for strained silicene starting from the four-orbital model in which the crystal lattice distortions under strain were accounted for through the Slater-Koster hopping integrals. We reduce the full four-orbital Hamiltonian to a single-orbital tight-binding Hamiltonian, linearized in ${\vec k}$ close to the Dirac points, which is useful for subsequent spin accumulation calculations under a small electric field. This approach can also be extended for other two-dimensional materials such as monolayer $\hbox {MoS}_2$^64^ and other graphene analogues such as phosphorene^65^ and stanene^66^.

Our derivation reveals terms in the Hamiltonian that have been neglected in previous studies on strained silicene. In particular, the breaking of the directional isotropy in strained silicene leads to the emergence of in-plane components in the spin–orbit interaction field near the Dirac points and an anisotropic energy dispersion relation in the vicinity of the Dirac points. As an exemplary illustration of the effects of these new terms, we studied the current-induced spin accumulation in strained silicene with an in-plane magnetization. The in-plane components of the spin–orbit interaction field at the Dirac points coupled to the in-plane magnetization lift the energy degeneracy between the two valleys. In combination with the anisotropy of the energy dispersions in strained silicene, we have the breaking of the anti-symmetry of the spin accumulation about the Fermi surface as well as between the two valleys. This results in a finite out-of-plane spin-*z* accumulation, which can be utilized in current-induced spin–orbit torque magnetization switching.

## Supplementary information

Supplementary Information.

## Data Availability

The datasets generated during the current study are available from the corresponding author on reasonable request.
