# Supplementary material for: Effective Hamiltonian for silicene under arbitrary strain from multi-orbital basis
Source: Sci Rep. 2021 Apr 7;11:7575. doi: 10.1038/s41598-021-86947-z (PMC8027682; doi:10.1038/s41598-021-86947-z)
Supplement: Supplementary file 1 — Supplementary Information. [file 41598_2021_86947_MOESM1_ESM.pdf]

Supplementary information

Effective Hamiltonian for silicene under arbitrary strain from multi-orbital basis

Zhuo Bin Siu and Mansoor B. A. Jalil
Department of Electrical and Computer Engineering, National University of Singapore, Singapore

SUPPLEMENTARY NOTE

The k-space position of the Dirac point in the first Brillouin zone is not captured by the low-energy Hamiltonian of equation (29) in the main text and it is unimportant in the resultant low-energy physics of the homogenous systems considered in this work. However, the relative k-space and energy displacements of the Dirac points in different segments of *inhomogeneous* systems such as a heterojunction between strained and unstrained silicene does affect, for example, the transmission across the heterojunction. Hence, we plot the loci of Dirac points for the positive-k_x Dirac valley, corresponding to the strained system described by equation (31) in the main text as the strain angle α is varied. We consider the unstrained silicene case ($\gamma = 1$), as well as strained silicene with $\gamma = 1.01$ and $\gamma = 1.1$, as shown in Fig. 1. Figure S1(a) shows that the loci of the Dirac points move around an approximate ellipse in k-space

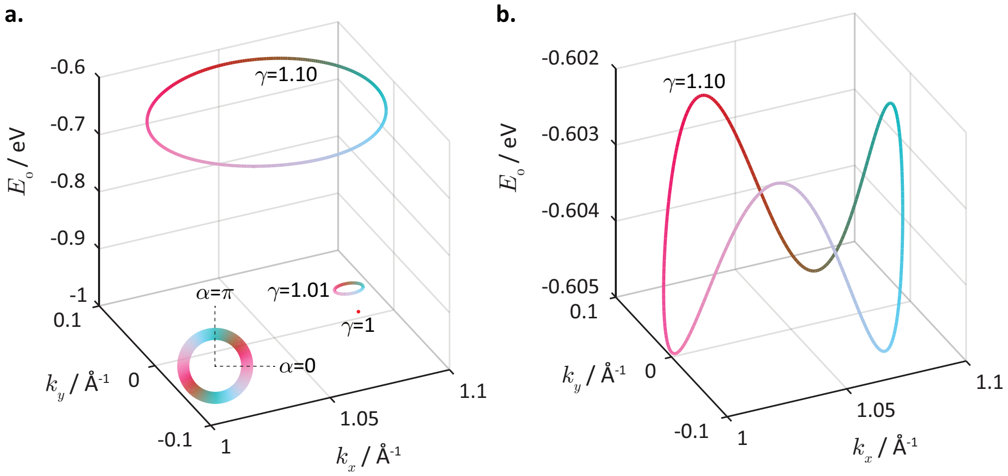

Figure 1. Loci of Dirac points in the positive-k_x valley in energy and k-space. (**a**) shows the loci for unstrained silicene ($\gamma = 1$), and strained silicene with $\gamma = 1.01$ and $\gamma = 1.1$, with the variation of the strain angle α, as indicated by the color wheel. Note that strain angles of α and $(\alpha + \pi)$ are physically equivalent to one another. (**b**) is a zoomed-in view of the locus of Dirac points for the case of $\gamma = 1.1$.

as the strain angle is varied. Panel (b) of the figure shows that the energy of the Dirac point varies slightly with the strain angle and has three peaks and troughs that result from the approximate three-fold rotational symmetry of the strained silicene lattice. (The rotational symmetry is only approximate and not exact because of the strain-induced lattice distortion.) The mean energy and k-space centroid of the elliptical loci of Dirac points are both displaced from the energy and k-space position of the Dirac point of unstrained silicene. The extent of the displacements increases with the strain magnitude, as can be seen by comparing the $\gamma = 1.01$ and $\gamma = 1.1$ loci in panel (a) of the figure.
